# The Validation of a Non-Invasive Skin Sampling Device for Detecting Cetacean Poxvirus

**DOI:** 10.3390/ani11102814

**Published:** 2021-09-27

**Authors:** Simone Segura-Göthlin, Antonio Fernández, Manuel Arbelo, Idaira Felipe-Jiménez, Ana Colom-Rivero, Javier Almunia, Eva Sierra

**Affiliations:** 1 Veterinary Histology and Pathology, Atlantic Center for Cetacean Research, University Institute of Animal Health and Food Safety (IUSA), Veterinary School, University of Las Palmas de Gran Canaria (ULPGC), Trasmontaña, s/n, 35413 Las Palmas, Spain; siimone.andrea@gmail.com (S.S.-G.); manuel.arbelo@ulpgc.es (M.A.); idaira.fjvet@gmail.com (I.F.-J.); acolomrivero@gmail.com (A.C.-R.); eva.sierra@ulpgc.es (E.S.); 2 Loro Parque Foundation, Avda. Loro Parque, s/n, 38400 Puerto de la Cruz, Spain; dir@loroparque-fundacion.org

**Keywords:** cetacean poxvirus, skin lesions, health indicator, welfare, biopsy, cytology cell sampler, DNA extraction, PCR, cetaceans

## Abstract

**Simple Summary:**

The current growing social awareness of animal welfare has led to the development of welfare indicators, which are effective tools for assessing each of the integrated aspects of this multidisciplinary issue. Hence, skin diseases have been suggested as potential general health indicators for use in cetaceans. Particularly cetacean poxvirus causes distinguishable hyperpigmented “ring” or “tattoo” lesions that affect cetaceans both in the wild and in managed facilities. However, most studies have analyzed these characteristic lesions through visual appraisal, while only a few have implemented diagnostic methods to corroborate the presence of the virus. To this end, skin biopsies are usually the sampling method selected, although they are considered to be an intrusive procedure. In this study, we analyzed sloughed skin sampled with cytology cell samplers (CCSs) in 12 tattoo-like lesions from two free-ranging cetaceans stranded in the Canary Islands. We employed two different DNA extraction methods and compared the effectiveness of the device with that of biopsies. All the lesions resulted positive for cetacean poxvirus, obtaining reliable data from the use of this device. Thus, CCS is considered to be a promising non-invasive tool for further assessing skin diseases in cetaceans, particularly those under human care, without affecting their welfare.

**Abstract:**

Poxvirus-like lesions are widely used as a potential health indicator in cetaceans, although for this application, corroboration of Poxvirus skin disease is imperative. Aiming to address skin biopsies intrusiveness, a preliminary investigation of a non-invasive skin sampling procedure to molecularly detect CePV-1 in 12 tattoo-like-lesions from two free-ranging stranded cetaceans in the Canary Islands was performed. Skin lesions were brushed with cytology cell samplers (CCSs) and placed into 1.5 mL microcentrifuge tubes with 1 mL of RNA*later*^TM^ Stabilization Solution. For factual comparisons, DNA extractions from sloughed skin obtained with CCS and biopsies from the same lesions were accomplished with DNA Tissue Kit S^TM^ (QuickGene, Kurabo, Japan). Moreover, a second DNA extraction from sloughed skin with DNeasy^TM^ Blood and Tissue Kit (Qiagen, Inc., Valencia, CA, USA) was performed to ascertain kit suitability for CCS. Molecular detection of CePV-1 was performed through a real-time PCR. As a result, a 91.7% and 83.3% rates of positivity were obtained with biopsies and CCS through Quickgene, respectively, compared to the rate of 100% using CCS with Qiagen. Accordingly, CCS is a reliable non-invasive sampling device to obtain sufficient genetic material to be analyzed for CePV-1 in tattoo-skin-lesions as well as for other purposes in cetaceans under human care.

## 1. Introduction

Cetaceans’ skin is considered a multidimensional feature that can provide a wealth of information, forming the basis of research in a wide number of studies covering a broad range of scientific branches [1,2,3,4,5,6,7,8]. Hence, this tissue has been used for long-term health assessments, enabling us to gain a closer look at the health status of marine mammals and aquatic ecosystems [9,10,11,12]. For instance, skin diseases have been suggested to be triggered by exposing both free-ranging and human-managed cetaceans to continuous aberrant conditions, resulting in compromised immune system function and a consequent increase in susceptibility to disease [13,14,15,16].

Cetacean poxvirus (CePV) is one of the most widely reported skin diseases [17,18,19,20,21]. Recently, it has been classified into two groups: cetacean poxvirus 1 (CePV-1), which affects both free-ranging and human-managed odontocetes, and cetacean poxvirus 2 (CePV-2), which infects mysticetes [22,23]. In cetaceans, this cutaneous disease displays characteristic lesions which are recognized as hyperpigmented “ring” or “tattoo” lesions, with the latter being referred to as tattoo skin disease (TSD) [20]. Regarding the unanimous consensus that clinical signs of disease can be indicative of compromised health, these distinguishable skin manifestations have been considered as a potential general health indicator in cetaceans [13,24,25]. Despite being broadly described, this viral skin disorder is still unassigned within the *Chordopoxvirinae* subfamily due to the limited genomic information on it. One of the main reasons for this is that CePV has mainly been identified through visual appraisal [25,26,27,28], with few studies having used diagnostic assays to correctly detect and therefore determine the presence of this pathogen in poxvirus-like lesions in cetaceans [22,24,29,30,31].

The detection of CePV in tattoo-like lesions in cetaceans under human care is necessary in order for these lesions to be applied as an “animal-based” health indicator [32,33,34,35]. As most research fields in which skin is the focus of study, skin biopsies are the method of choice to either molecularly or histologically diagnose skin diseases in cetaceans in managed facilities [36,37,38,39,40,41]. Nevertheless, the increasing awareness of welfare in cetaceans has prompted attempts to develop dynamic methodologies for safe handling and sampling with the aim of minimizing the risk of compromising their well-being. As with wild individuals, in managed facilities some researchers have highlighted the fast turnover time of cetaceans’ skin [42,43,44,45,46], proposing the collection of sloughed skin of animals’ bodies as an advantageous non-invasive method that could potentially be an alternative to biopsies [5,47,48]. Notwithstanding the aforementioned points, research on the use of these emerging non-intrusive skin sampling techniques in cetaceans under human care is still scarce, with their effectiveness having been poorly explored in research studies.

Correspondingly, as managed facilities are actively committed to the advancement of scientific research while maintaining ethical responsibility, efforts to create innovative sampling methodologies and improve the standards of practice during these procedures should be encouraged [49,50,51]. Thus, stranded cetaceans could plausibly be used in model studies, providing an opportunity to perform preliminary investigations [52,53,54]. Additionally, their use would enable protocol adjustments to resolve possible misgivings and achieve feasible results that could be reproduced in cetaceans under human care.

Accordingly, the aim of the present study is to validate a potential non-invasive skin sampling device using sloughed skin to molecularly detect cetacean poxvirus (CePV) in tattoo-like lesions by comparing its sensitivity and effectiveness with that of skin biopsies obtained from stranded cetaceans in the Canary Islands.

## 2. Materials and Methods

**Figure 1:**
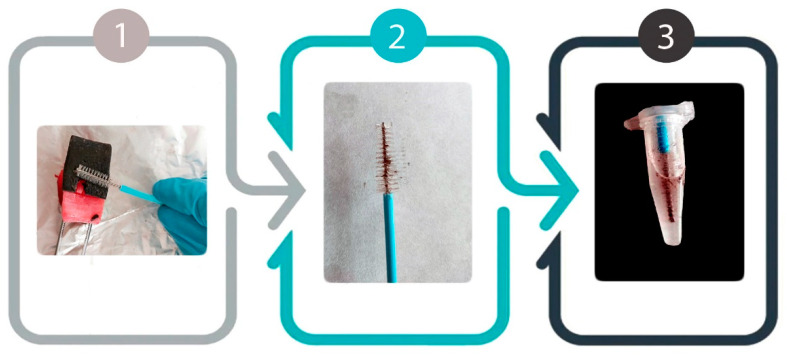
Workflow illustration of the cytology cell sampler skin sampling methodology.

On 21 February 2021, a juvenile male common bottlenose dolphin (*Tursiops truncatus*) (Case 1), 240 cm in length, was found stranded and dead at Abades, Arico, Tenerife, Canary Islands, Spain (28°09′00″ N, 16°25′00″ W). On 17 April 2021, a juvenile female Atlantic spotted dolphin (*Stenella frontalis*) (Case 2), 150 cm in length, was found stranded and dead at Playa San Juan, Guía de Isora, Tenerife, Canary Islands, Spain (28°10′47″ N, 16°48′45″ W). Based on their anatomic parameters, both animals showed a moderate body condition [55] and the carcasses were in a good state of preservation (code 2/5) [56,57,58,59]. Due to their exceptional states of preservation, neither the refrigeration nor freezing of either of the animals were required prior to their necropsies. Thus, standardized necropsies [60] were performed on each dolphin the day after they were found. Throughout the external examination during necropsies, several skin lesions affecting the rostral and lateral areas of both animals were observed. As a result, two lesions from Case 1 and ten lesions from Case 2 were described, photographed, and measured before their later collection. Each skin lesion from both animals was split to retain a portion at −80 °C, while the remainder was first sampled with a sterile cytology cell sampler (CCS) (Deltalab, Barcelona, Spain) and later correctly identified and preserved at the same temperature as the other portion. The skin sampling procedure using these CCSs consisted of gently brushing the surface of the lesions to obtain sloughed epidermis, which adhered into the bristles of the brush. Then, all the CCSs were placed into 1.5 mL sterile RNAse- and DNAse-free microcentrifuge tubes with a safe lock (Thermofisher Scientific, Madrid, Spain), in which 1 mL of RNA*later*^TM^ Stabilization Solution (Thermofisher Scientific, Madrid, Spain) had previously been added. Subsequently, the bristles of the CCS stayed embedded in the RNA*later*^TM^, while the plastic stems were cut to the level of the microcentrifuge tubes’ tops with a pair of scissors to allow the closure of the vials, using the safe lock to avoid unexpected openings (Figure 1). Due to the genomic stabilization capacity of the *RNAlater*^TM^ solution, microcentrifuge tubes were stored at room temperature until their subsequent molecular analysis, which was performed within 1 working week [61].

After accomplishing the procedure previously explained, the rest of both necropsies were performed by sampling and collecting representative tissues of all the major organs and lesions for subsequent analyses in order to proximate the most plausible cause of death/stranding, as routinely performed [58,59]. Hence, all samples were stored in a 10% neutral buffered formalin fixative solution for histologic and immunohistochemical analysis, whilst few of them were preserved at −80 °C until processing for biomolecular studies.

Approximately 0.5 g of each fresh-frozen skin samples from both animals was mechanically macerated in lysis buffer and subsequently centrifuged, later progressing to simultaneous DNA/RNA extraction using the DNA Tissue Kit S^TM^ (QuickGene, Kurabo, Japan). Considering that an initial sample of ≤0.5 g is required to correctly perform genomic extraction with this method, some modifications in the manufacturer protocol were necessary in order to accurately extract the DNA/RNA from the fresh skin samples collected with CCS. They were first agitated using a vortex for 15 s at maximum speed to ensure the detachment and mixture of a great part of the epidermal crust adhered among the bristles into the 1 mL RNA*later*^TM^ solution. After this, the tips of the CCS were removed, preserving the acquired RNA*later*–sloughed skin mixture in the vials. With the aim of obtaining an approximate amount of 5000 μL of macerates from each sample, some adaptations in the proportions of the components were made. Therefore, instead of adding 4500 μL of 0.1% diethylpyrocarbonate (DEPC)-treated water and 500 μL of 1× lysis buffer as accomplished with biopsy samples, 3600 μL and 400 μL from each component, respectively, were applied apart from the 1000 μL RNA*later*–sloughed skin mixture. Finally, all macerates were centrifuged (2500 rpm for 15′ at 4 °C) and supernatants were collected to continue with their genomic extraction. DNA/RNA extraction was achieved from each macerated sample (N = 24) in a QuickGene Mini 80 nucleic acid isolation machine (QuickGene, Kurabo, Japan) according to the manufacturer’s instructions with some modifications: an RNA carrier (Applied Biosystems^TM^, Thermo Fisher Scientific, Waltham, MA, USA) was added during the lysis step, as previously indicated [62].

The molecular detection of CePV-1 was performed using a 1-step real-time polymerase chain (q-PCR) method to amplify a conserved region (150 bp) of the DNA polymerase gene by using the degenerate primer sets designed by Sacristán et al. [63] (Odontopox-F: 5′-CARGAAATMAAAAAGAARTTTCCATC-3′, and Odontopox-R: 5′-ACGTTCTGTTAARAAYCGTCTTAGTA-3′). The thermocycler profile was set for initial denaturation at 95 °C for 5 min, followed by 40 amplification cycles, each compromised of a denaturation step at 95 °C for 15 s, an annealing step at 60 °C for 30 s, and an elongation step at 72 °C for 30 s. The final cycle was composed of an extended elongation, which was performed at 72 °C for 7 min [29]. A melting curve step was added at the end of the reaction. The thermal cycler employed was a MiniOpticon^TM^ Real-Time PCR System (Bio-Rad Laboratories, Irvine, CA, USA). Adequate non-template negative controls (nuclease-free water) for both extraction and amplification as well as extraction-positive and amplification-positive controls previously confirmed by our group were included.

The PCR products from positive lesions were purified using a commercial kit (Real Clean Spin kit 50 Test-REAL), and then sequenced using Sanger DNA sequencing (Secugen S.L., Madrid, Spain). The amplicon identities were confirmed with BLAST (www.ncbi.nlm.nih.gov/blast/Blast.cgi/ (accessed on 4 June 2021)).

In order to compare the effectiveness of the DNA extraction from the skin samples collected with CCS using the QuickGen kit method, a second extraction using DNeasy^TM^ Blood and Tissue Kit (Qiagen, Inc., Valencia, CA, USA) was undertaken. In this instance, it was necessary to unfreeze each of the halves which had previously been scraped with CCS from the 12 skin samples collected from Cases 1 and 2. Hence, the same skin sampling procedure using CCS explained above was performed repeatedly. The CePV-1 positive control was a 0.025 g biopsy that had previously been confirmed by our group using the real-time PCR method [63] and sequencing amplicons (unpublished sequencing results) previously described. Once 1 mL RNA*later*–epidermis mixtures were obtained, they were subsequently subjected to a high centrifugation speed for 5 min (14,000 rpm). On some occasions, this step had to be repeated as many times as necessary to obtain enough pellet. Consequently, approximately 0.025 g of skin pellet precipitation was collected from each sample by removing practically all the supernatant. To verify that the maximum sample weight specified by the manufacturer’s instructions for correctly performing the DNA extraction had not been exceeded, all the vials were weighted. Thus, precise weights of samples were acquired by subtracting the weight of an empty vial (≈1.078 g). Afterwards, all samples were ready for the DNA extraction to proceed following the manufacturer’s instructions. The importance of incubating the biopsy CePV-1 positive control, which had previously been cut into small pieces, for at least 30 min with continuous 15 s high-speed vortexing every 5 min during incubation for complete lysing must be noted. Upon the completion of the extraction, DNA products were tested with the same real-time protocol as that mentioned above (see Appendix A for more details). 

## 3. Results

### 3.1. Macroscopic Findings

#### 3.1.1. Case 1

**Figure 2:**
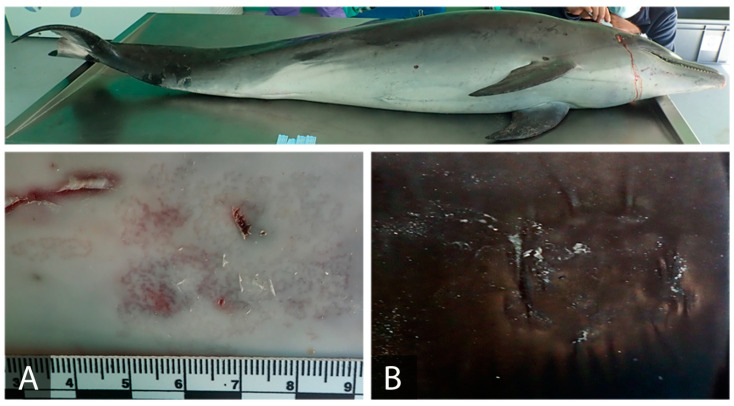
Gross lesions compatible with CePV in bottlenose dolphin, Case 1. Right lateral view. (**A**) Irregular, stippled, and serpiginous grey lesion (5 × 3.5 cm) located ventral to the right corner of the oral cavity. (**B**) Oval and depressed lesion (2.5 × 2 cm) on the right lateral side of the melon.

In total, Case 1 (Figure 2) showed two lesions that could be attributable to CePV. Of both lesions, the most remarkable was a 5 × 3.5 cm serpiginous and stippled light grey tattoo-like lesion located on the ventral right corner of the oral cavity (Figure 2A). The other lesion (Figure 2B) showed an oval and depressed shape, which was observed on the melon of the common bottlenose dolphin.

#### 3.1.2. Case 2

**Figure 3:**
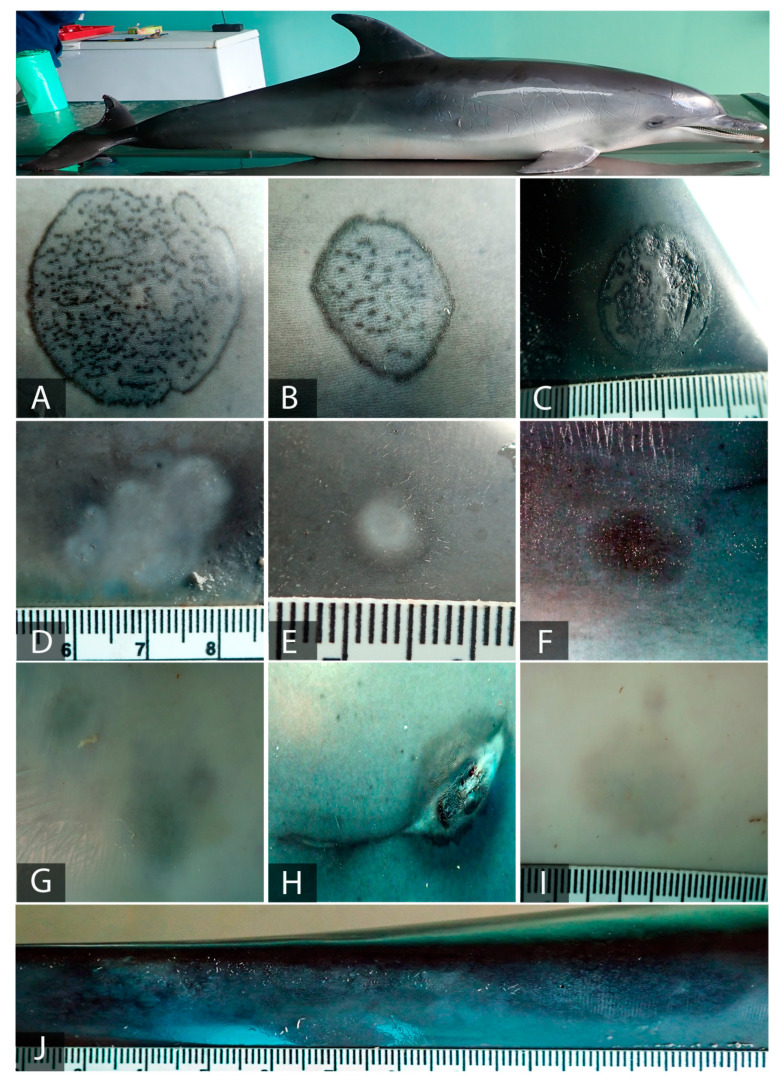
Gross lesions compatible with CePV in Atlantic spotted dolphin, Case 2. Right lateral view. (**A**) Ring lesion with a black edge and stippled pattern center (3 × 2.3 cm) on the right side of the melon. (**B**) Ring lesion with a black edge and stippled pattern center (1 × 0.7 cm) located on the right side of the melon. (**C**) Oval lesion presenting both margin and inner ping-hole pattern slightly raised with half of the center blistered (1.8 × 1.3 cm), located on the right side of the dorsal fin. (**D**) Irregular pale and coalesced wound with a barely visible dark edge (2.3 × 1.2 cm) located on the right dorsolateral superior hemimaxilla. (**E**) An oval lesion with a pale center and blurred margin (0.6 × 0.3 cm) situated on the right dorsal part of the tip. (**F**) Oval dark lesion with pale margin (1.5 × 1 cm) located on the right side of the animal. (**G**) A blurred and irregular grey lesion on the rostroventral part. (**H**) An oval dark lesion with a pale, raised, and irregular center (1.6 × 1.3 cm) on the right lateral side. (**I**) A blurred hardly visible grey lesion (1.8 × 1.5 cm) on the ventral part of the animal. (**J**) A large and irregular dark lesion with a greyish pin-hole pattern across the entire center located on the dorsal part of the peduncle.

Just like Case 1, Case 2 (Figure 3) presented compatible CePV lesions. In a multifocally manner, tattoo-like lesions with different evolution stages were randomly distributed and affected many areas of the skin. Three of them (Figure 3A–C) were the characteristic persisting ring lesions, delimited with black edges and showing a black and stippled pattern at the center. One of these lesions (Figure 3C) showed blistering across half of its center. Another two lesions (Figure 3D,E) were observed on the tip and melon of the dolphin, respectively. They were lighter in color and featured a barely visible black margin, corresponding to the lesions in the healing process. On one of the flanks of the spotted dolphin, a ring lesion that was black in color with pale edges was observed (Figure 3F). Close to it, a lesion that appeared very similar to this last one, apart from its pale, irregular, and raised center, was observed (Figure 3H). The lesions observed at the ventral part of the animal (Figure 3G,I) were irregular, light grey, and blurred, being hardly perceptible and without delimiting margins. On the peduncle, there was a remarkably large lesion affecting almost all the entire length (Figure 3J). This lesion was irregular in shape and black, and featured a pale grey pin-hole pattern along its center.

### 3.2. Molecular Findings

The results of the molecular findings are compiled in Table 1. Of the 12 cutaneous lesions sampled using biopsies taken from both individuals, which were previously submitted for Quickgene DNA/RNA extraction, 11 were positive for CePV-1. More specifically, from Case 1, both lesions were positive; meanwhile, in Case 2, of the 10 lesions tested, nine were positive. Only one lesion (Figure 3J) presented an abnormal amplification curve with a RT-PCR cycle threshold value (Ct) of 11.66 without melting temperature. For the purpose of confirming this lesion as negative to CePV-1 and to prove that the PCR product was neither too concentrated nor overloaded with inhibitors leading to incorrect PCR interpretations, it was diluted into 10-fold serial dilutions up to 10^−3^. In this way, we sought to stablish a better sensitivity and quantification dynamic range. However, the real-time PCR detected neither of the dilutions of the PCR product from this lesion.

Regarding the samples collected with sterile CCS from these 12 lesions, different results were obtained depending on which genomic extraction method was used. Hence, with Quickgene, 10 lesions from both cases were found to be CePV-1 positive: both lesions from Case 1 (Figure 2A,B) and eight lesions from Case 2 (Figure 3A–F,I,J). Comparing these results with those obtained with tissue sampling, it can be observed that, in both sampling methods, the same lesions from Case 1 were found to be positive for cetacean poxvirus. Nevertheless, the same outcome was not observed in Case 2, in which a lesion that was found to be negative when sampled with a biopsy (Figure 3J) was found to be positive when sampling using CCS. However, using this latter sampling method, two other lesions which were found to be positive for CePV-1 when collected using a biopsy (Figure 3G,H) were not detected. Conversely, through Qiagen, all lesions from Case 2 were found to be positive for poxvirus, in addition to the other two lesions from Case 1. Therefore, making a general comparation from these results with the other obtained by employing a different genomic extraction method, we observed that using the same sampling procedure (CCS), the two lesions that were not detected (Figure 3G,H) were both found to be positive with Qiagen. Moreover, with this last genomic extraction kit, the negative tissue sample from Case 2 was also found to be positive when CCS was employed. Thus, from a broad-based assessment, we could observe that with Quickgene, a slightly better sensitivity was acquired when samples were collected with biopsy rather than with CCS. Yet, an improvement on these results was gained when applying both the CCS sampling method and the Qiagen extraction kit.

Comparing the Ct values from positive lesions between both different sample collections and genomic extraction methods a remarkable range of the Ct values was observed, with 15.17 and 37.10 being the minimum and the maximum values, respectively. Considering the theoretical correlation in which it was established that low Ct values correspond to a high viral loads and vice versa, it is observed that the lesion 3A from Case 2 presented the maximum viral load in both sampling techniques and extraction methods. In addition, the same sigmoidal correlation was observed between the lesions that had the lowest viral load extracted with Quickgene. Thus, lesion 3H, which was sampled by biopsy, had a Ct value of 35.37, with it being undetected when using the cytology cell sampler. However, these results did not concur with the ones obtained with Qiagen, with lesion 3G being the one which presented the least viral load, with a Ct value of 37.10.

The sequence similarity searching from the DNA polymerase sequences of CePV-1 obtained from all the positive lesions of both cases in this study was performed with BLAST. We could identify that, in both cases, the sequences revealed high percentage homologies of 100%, 99%, and 98% with the already uploaded nucleotides sequences under the GenBank accession numbers of MF458199, KU726612, and MH005249, respectively.

## 4. Discussion

### 4.1. Sampling Methods and DNA Extraction Protocols

In the present study, the molecular detection of cetacean poxvirus from two free-ranging cetaceans stranded in the Canary Islands was achieved, with different results being obtaining depending on the sampling method used and the genomic extraction kit employed.

Aiming to reproduce and extrapolate the respective sampling procedures used for cetaceans under human care, skin samples were attained in fresh conditions. The use of sterile CCS enabled us to obtain an acceptable quantity of sloughed skin from all poxvirus-like lesions for posterior genomic extractions. Macroscopically, the load of sloughed skin that adhered to the bristles of CCS was determined by the size of the lesions, with it being possible to gain more epidermal crust from larger samples than was possible from smaller ones. This resulted in it being easier to obtain epidermal crusts from lesions with a larger volume due to it being easier to rub their surfaces than ones with a smaller size, with it being necessary in the latter to scrape the outer layer more times to gather sufficient desquamating epidermal debris. Accordingly, the time required to finally acquire sloughed skin was found to be approximately 1 min in all attempts. Nevertheless, as was recently reported by Bechshoft et al. [5], enough skin cannot always be obtained from scraping alongside the flanks of bottlenose dolphins in managed care when using a rubberized scraper. This may be due to the high metabolic and mitotic activity which affected skin undergoes, leading to a continuous removal of epidermis, in contrast to healthy skin [17,18]. In either case, the variation in the quantity of sloughed skin obtained between each sample did not lead to further complications for the DNA extraction, as each of the protocols was standardized.

Since biopsy is the current method of choice for collecting skin samples, we decided to compare the genomic yield gained from this sampling method with that obtained through CCS by using a DNA extraction kit that was specifically suitable for use with tissue samples [64]. Therefore, through Quickgene, we attempted to contrast the reliability and effectiveness of both sampling procedures in order to gain enough genetic material from the skin lesions.

Furthermore, Qiagen was used for a second DNA extraction from sloughed skin obtained from the same poxvirus-like lesions. According to the manufacturer’s protocol, Qiagen is suitable for purifying DNA from very small amounts of starting material, ensuring high-quality yields from nonstandard samples and considering 0.025 g as the maximum weight [65]. Therefore, the purpose of this second genomic extraction was to corroborate the point mentioned above, comparing the genome extraction from the sloughed skin that was collected via CCS through both kits. Thus, this study not only attempted to show which of the two sampling methods obtained more sensitive results, but also attempted to ascertain which of the genomic extraction protocols is more appropriate for use with the proposed non-invasive sampling method. In contrast to the first extraction, which was carried out through Quickgene, the undeliberate unfreezing of skin lesions from both animals had to be conducted to repeat the skin sampling procedure with CCS. This feature is recognized to have an undesirable impact on the quality of DNA preservation, apart from not serving as a standard operating procedure if it is intended to be extrapolated in cetaceans kept in managed facilities. Despite this, the DNA extraction was carried out while considering the latter facts regarding the further interpretation of the molecular results. 

In both the CSS sampling procedures, the sloughed skin was embedded in RNAlater^TM^ Stabilization Solution. The purpose of the use of this reagent is that it can serve as a transport medium in situations in which samples cannot be immediately processed or frozen, as often happens in managed facilities, where samples are normally sent to external laboratories.

### 4.2. q-PCR Molecular Results from CePV-1 Positive Lesions

The visual diagnosis of TSD was confirmed in all the samples tested in the present study. Nevertheless, the results differed when using the different tissue sampling methods and DNA extraction kits.

Focusing on the juvenile bottlenose dolphin (Case 1), both lesions were found to be positive when employing both sampling and genomic DNA extraction protocols. Lesion 2A presented a typical serpiginous irregular pattern and was delimited with black borders. In the literature, these lesions are considered to represent the acute phase of infection [24]. On the other hand, the other lesion, 2B, would have been hard to detect if it were not for its depressed and oval-shaped appearance. This lesion is considered to be in an advanced stage of the infection [18]. Through Quickgene, it can be observed that both the biopsy and CCS sampling methods were effective for both lesions. However, it is evident that lesion 2A showed a higher viral load than lesion 2B, with the biopsy sample showing a better Ct value (23.65) than the sloughed sample (25.46). Nevertheless, both values indicate a considerable viral load when in terms of poxvirus infections. Thus, there is a correlation between the macroscopic findings, since 2A was considered to be in an initial stage and due to the viral load. Regarding the other positive lesion, 2B, it was also successfully extracted using both sampling methods. However, in this case it was the sloughed sample that presented a better Ct value (27.93) compared to the biopsy (31.80). In addition, these molecular results are also correlated with the advanced stage of the lesion. The Qiagen extractions of sloughed skin collected from both lesions gained good DNA genomic yields, to such an extent that each lesion presented even better Ct values than those found using the Quickgene extraction, with Ct values of 20.85 and 26.70, respectively.

In the Atlantic spotted dolphin, different evolution stages of 10 tattoo-like lesions were observed, coinciding with a wide range of Ct values. Macroscopically, the first three lesions (3A–C) were typical rounded lesions with a stippled pattern in the center, representing the early stage of the infection [24]. Lesions 3A and 3C were larger in size than 3B and also presented considerably more dark pinpoints at the center. Moreover, lesion 3C presented slightly raised margins and half of its center was blistered; both features could be used to identify acute phases of the infection [18]. Regarding the Quickgene biopsy genome extractions, the Ct values obtained from these three tattoo-like lesions were 15.65, 18.08, and 16.42, respectively, with these lesions having higher viral loads than all the others tested in the present study. The same pattern can be observed in the molecular results from the CCS, with Ct values of 17.25, 20.80, and 19.04. In this case, the biopsy samples gathered better genomic yields than the sloughed samples when using same extraction kit. Concerning the DNA extraction yield with Qiagen from the sloughed skin, very similar results are obtained. Regarding these first three lesions, in this case the biopsy samples gained better Ct values from 3A and 3B, with only the result for the third lesion, 3C, being improved with the use of Qiagen.

Within the other seven lesions from specimen 2, the variations in molecular values between the sampling methods through Quickgene were significant. At first glance, the lack of CePV amplification on the three lesions can be noticed. Regarding 3J, we were not able to amplify poxvirus DNA using the biopsy sample. Conversely, the same lesion was amplified when using the CCS sampling technique. Our first impression was that the PCR product from the biopsy sample was overloaded, leading to amplification faults. However, once they were diluted into serial dilutions, none of them were found to be positive for CePV. These results might suggest that an inappropriate genomic DNA extraction procedure was used for this lesion, or that incorrect sampling was achieved due to selecting an area from the lesion without viral content. Whatever the case, the Ct value from the sampling of this lesion with CCS was 33.20, indicating a low viral load, a feature which might have also influenced the result obtained with the biopsy. The other two lesions from which DNA was extracted that did not have amplified poxvirus sequences were 3G and 3H. In this case, both lesions were sampled with CCS. This might have been due to their low viral loads (the samples presented values of 31.79 and 35.37 from biopsy sampling, respectively), meaning that they were undetected when collected from sloughed skin. On the other hand, and corroborating the above point, lesion 3G was barely visible macroscopically and featured a dark-grey area, appearing to be an almost healed tattoo-like lesion [33]. However, contrary to what might be expected, lesion 3H, which was compared to 3G in a prior evolution stage, presented a lower viral load than the other lesion. When analyzing the molecular results obtained through Qiagen for these three lesions, it is possible to reach more reasonable conclusions. The DNA extracted from sloughed skin from lesions 3G, 3H, and 3J using this kit were found to be positive for CePV-1. The Ct values obtained were 37.10, 35.66, and 31.88, respectively. In this case, an expected clear correlation between the low expression level of Ct values and macroscopical findings can be observed. Interestingly, the Ct results for lesion 3J were more favorable when using this genomic extraction protocol employing CCS as a sampling method. Accordingly, as has been reported, Ct values of above 35 in q-PCR are not considerable and should not be interpreted as marginally positive. However, the melting curves obtained from these PCR products were identical for all positive samples, and negative controls did not produce any product. Due to this, they were sequenced to confirm their specificity, leading to poxvirus DNA polymerase sequences being obtained.

The rest of the lesions (Figure 3D–F,I) were all in different stages of regression, with black margins being less evident or disappearing and the lesions becoming lighter in color [20]. The four of them presented low viral loads when using both genome DNA extractions, with slightly better Ct values being obtaining with the biopsy compared to with CCS through Quickgene. The other genomic extraction protocol obtained barely weakened molecular results compared with both sampling methods.

### 4.3. Validating Cytology Cell Samplers as a Reliable Non-Invasive Method to Sample Skin Lesions

In attempting to determine the effectiveness of the CCS, different percentages of positive results were obtained when comparing the sampling and genomic extraction methods. In this manner, considering that all 12 lesions were determined to be CePV-1 positive through Quickgene, the effectiveness of detecting this virus in skin lesions was 91.7% and 83.3% when using biopsy and CCS, respectively. From this, it can be deduced that sampling with skin biopsies has an 8.4% accuracy. Comparing the Ct values of both sampling methods when using Quickgene as a genome extraction kit, it is evident that the lesions sampled with CCS require more amplification cycles in order to cross the positivity threshold. This is reflected in the negative Ct values of the lesions sampled with CCS (Figure 3G,H). Accordingly, as mentioned before poxvirus lesions in healing stages with low viral loads might lead to CCS losing a certain amount of sensitivity. However, these results could be improved by the use of Qiagen with CCS, which had a 100% success rate. 

Considering physical status as an important aspect of an animal’s overall wellbeing, detecting cetacean poxvirus in tattoo-like lesions is important in order to correctly corroborate an animal’s condition. Hence, such characteristic lesions could generally serve as an indicator of disease progression, thus correlating them with the health state of the animal. In cetaceans under human care, the presence of confirmed poxvirus lesions could potentially be used as a visual health parameter, especially when combined with the advantage of applying CCS as a non-invasive skin sampling procedure which is unlikely to negatively interfere with the welfare of animals.

Having access to two specimens with positive poxvirus lesions was very significant in our quest to validate CCS as an effective viral skin sampling method. The exceptional states of preservation of the animals was crucial in developing the present skin sampling protocol in order to be used for cetaceans under human care. In addition, despite the limitations of the sample size, the fact that all 12 lesions were found to be positive for cetacean poxvirus through CCS was outstanding and reaffirms the need to prove their efficacy in cetaceans under managed care.

In summary, this pilot study on stranded animals has served as an opportunity to validate the use of sterile CCS for the diagnosis of poxvirus skin disease. The skin sampling procedure making use of sterile CCS can be considered to be a promising method for the detection of cetacean poxvirus, with accurate results for animals in managed care. Furthermore, we can additionally consider their implementation for sampling sufficient genetic material for other multiple areas of study, not limiting their applicability in poxvirus skin disease. Additionally, this leads to the idea that there should be a rigorous discussion as to whether biopsies are truly the best sampling method for detecting pathogenic microorganisms such as viruses in cetaceans under human care. This needs to be balanced against the potential stress and risk caused to the individual by the handling and sampling processes. However, further investigation is needed to address the uncertainties involved and ensure the potential of the use of this non-invasive method in cetaceans in managed facilities.

## 5. Conclusions

In the present study, we demonstrated the reliability of the use of CCS for the detection of cetacean poxvirus, comparing the results with those of biopsy samples. These findings will be highly significant for validating the further use of this device as a non-invasive method for assessing viral skin lesions in cetaceans under human care and carrying out visual health assessments.

## Figures and Tables

**Table 1:** Molecular results from tissue and cytology cell sampler sampling methods using both the DNA Tissue Kit S^TM^ (QuickGene, Kurabo, Japan) and the DNeasy^TM^ Blood and Tissue Kit (Qiagen, Inc., Valencia, CA, USA) on skin lesions from a bottlenose dolphin and an Atlantic spotted dolphin stranded on the Tenerife coasts, Canary Islands, Spain, 2021.

Case	Lesion	Tissue		Cytology Cell Sampler			
		QuickGene		QuickGene		Qiagen	
		CePV	Ct	CePV	Ct	CePV	Ct
**1**	A	+	23.65	+	25.46	+	20.85
	B	+	31.80	+	27.93	+	26.70
**2**	A	+	15.65	+	17.25	+	17.14
	B	+	18.08	+	20.80	+	19.58
	C	+	16.42	+	19.04	+	15.17
	D	+	33.63	+	34.45	+	34.27
	E	+	25.02	+	33.55	+	34.74
	F	+	33.44	+	34.31	+	35.09
	G	+	31.79	−	n/a	+	37.10
	H	+	35.37	−	n/a	+	35.66
	I	+	28.49	+	33.33	+	31.97
	J	−	n/a	+	33.20	+	31.88

Notes: CePV-1, cetacean poxvirus; Ct, cycle threshold; Qiagen, DNeasy Blood and Tissue Kit (Qiagen, Inc., Valencia, CA, USA); QuickGene, DNA Tissue Kit S (QuickGene, Kurabo, Japan); +, positive; −, negative; n/a, not applicable.

## Data Availability

The present study does not report any data.

## Appendix A

As figured in the handling protocol, the DNA Tissue Kit S^TM^ (QuickGene, Kurabo, Japan) corresponds to genomic extraction from 0.5 g of animal tissue sample, making it ideal for genomic DNA/RNA extraction from the 12 poxvirus-like lesions biopsy samples used in the present study. Thus, 0.5 g of tissue from each skin lesion was used as a maximum amount of starting material. Regarding the samples collected with CCS, despite it being practically impossible to obtain the same quantity of sloughed skin from every skin lesion, adaptations were made in terms of the proportions of the maceration components. This resulted in there being 12 macerates with a final volume of 5000 μL, consisting of 1000 μL of RNAlater mixed with sloughed skin, 3600 μL of 0.1% DEPC-treated water, and 400 μL of 1× lysis buffer. In this manner, samples collected with CCS presented the same volume and did not broadly vary.

During the genomic extraction with the DNeasy^TM^ Blood and Tissue Kit (Qiagen, Inc., Valencia, CA, USA), prior to the lysis step, once all samples were centrifugated it was noticed that some of the vials did not show sloughed skin precipitations. On some occasions, repeated high-speed centrifugations were needed in order to achieve the decantation of the desquamating particles for further 0.025 g obtention. Despite this, DNA extraction from all sloughed samples could be conducted without some constraints. However, during the lysis step, extra time was needed for the positive control (0.025 g skin biopsy of a positive CePV-1 lesion from a short-finned pilot whale) in order to finally complete the lysis. Despite the small sample size and the fact that it was cut into even smaller pieces, the firmness of this tissue required the use of 30 min of incubation at 56 °C with continuous vortexing. Indeed, this is one of the reasons why we decided to carry out the genomic extraction of biopsy samples with Quickgene.
